# Exploring Adjunctive Novel Therapeutic Approach of KarXT (Xanomeline‐Trospium Chloride) for Managing Psychotic Symptoms in Patients With Schizophrenia and Alzheimer's Disease

**DOI:** 10.1002/brb3.71182

**Published:** 2026-01-15

**Authors:** Ashir Kanwal, Bismah Azeem, Hania Nasir, Fatima Mumtaz, Nauman Ashraf, Mohammed Hammad Jaber Amin, Warisha Kanwal, Bilal Wazir Khan

**Affiliations:** ^1^ Department of Internal Medicine Liaquat University of Medical and Health Sciences Jamshoro Pakistan; ^2^ Department of Internal Medicine Avicenna Medical College Lahore Pakistan; ^3^ Department of Internal Medicine Jinnah Sindh Medical University Karachi Pakistan; ^4^ Department of Psychiatry Ozark Center Joplin USA; ^5^ Department of Internal Medicine Alzaiem Alazhari University Khartoum North Sudan; ^6^ Department of Internal Medicine Karachi Medical and Dental College Karachi Pakistan; ^7^ Department of Internal Medicine Khyber Medical College, Peshawar Khyber Pakhtunkhwa Pakistan

**Keywords:** alzheimer's psychosis, KarXT, psychosis treatment, schizophrenia

## Abstract

**Background:**

Acute psychotic symptoms like delusions and hallucinations are of major concern while treating patients with schizophrenia and alzheimer's psychosis, primarily impacting their daily life functioning and quality of life. The traditional antipsychotic medications, commonly prescribed to manage these symptoms, cause significant side effects with limited efficacy, requiring novel therapeutic agents that can overcome this challenge. While there is no definitive cure, symptomatic treatment can help relieve some of the symptoms and improve the quality of life of people with alzheimer's disease (AD).

**Method:**

A comprehensive literature search of PubMed, scopus, google scholar, and ClinicalTrials.gov was conducted to identify studies on xanomeline–trospium chloride (KarXT) in schizophrenia and AD psychosis. After screening 802 unique records, 39 studies—including preclinical, clinical, and observational investigations—were included in this narrative review. Only English‐language publications up to February 2025 were considered.

**Result:**

KarXT, with its dual action on the M1 and M4 receptors and mAChR antagonism, greatly helps reduce the severity of the positive and negative symptoms, as it resulted in an 8.4‐point greater reduction on the PANSS scale. Side effects were minimal and did not account for the discontinuation of treatment.

**Conclusion:**

Psychosis is a common feature of schizophrenia and AD, most often caused by high concentrations of dopamine in the brain, characterized by hallucinations, delusions, and disorganized thinking, resulting in markedly reduced quality of life for the patient and associated caregiver. Conventional treatments targeting dopamine receptors produce extrapyramidal symptoms and metabolic side effects, leading to noncompliance with medication. KarXT, with its dual action on M1 and M4 receptors and mAChR antagonism, greatly helps reduce the severity of the positive and negative symptoms. The side effects experienced were minimal and did not account for the discontinuation of treatment.An overview of the mechanism of action, clinical trials, and classical findings of KarXT for the management of psychotic symptoms in patients with schizophrenia and alzheimer's disease.

## Graphical abstract

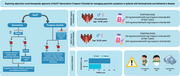

**Abbreviations**
(AChEI): Acetylcholinesterase Inhibitor
(AD): Alzheimer's Disease
(AEs): Adverse Events
(APA): American Psychiatric Association
(AUC): Area Under the Curve
(BBB): Blood‐Brain Barrier
(BID): Bis In Die (Twice Daily)
(BMI): Body Mass Index
(cAMP): Cyclic Adenosine Monophosphate
(Cmax): Maximum (peak) concentration of drug in plasma
(CNS): Central Nervous System
(D2R): Dopamine D2 Receptor
(DAG): Diacylglycerol
(DSM): Diagnostic and Statistical Manual of Mental Disorders
(FDA): Food and Drug Administration
(FDC): Fixed Dose Combination
(FGAs): First‐Generation Antipsychotics
(Gi/o): Gi/Go class of G proteins
(Gq): Gq class of G proteins
(IP3): Inositol Triphosphate
(KarXT) : Xanomeline‐Trospium Chloride
(mAChR): Muscarinic Acetylcholine Receptor
(NMDA): N‐Methyl‐D‐Aspartate
(NPI‐NH): Neuropsychiatric Inventory–Nursing Home version
(OLE): Open‐Label Extension
(PANSS): Positive and Negative Syndrome Scale
(RCT): Randomized Controlled Trial
(SGAs): Second‐Generation Antipsychotics
(TRRIP): Treatment Response and Resistance in Psychosis
(UTIs): Urinary Tract Infections
(Vd): Volume of Distribution
5(5‐HT2A): ‐Hydroxytryptamine (Serotonin) 2A receptor

## 1 Introduction

American psychiatric association's (APA) diagnostic and statistical manual of mental disorders defines psychosis as the presence of hallucinations, delusions, or both. Cognitive impairment and acute psychotic symptoms such as hallucinations, delusions, and disorganized thinking are features shared by several neuropsychiatric conditions, including AD and schizophrenia (Arciniegas 2015). When these symptoms occur secondary to another medical condition or substance use, they are classified as secondary psychotic disorders, which can include AD, Parkinson's disease, and diffuse Lewy body disease (American Psychiatric Association Task Force on DSM‐IV 2000).

AD is a progressive neurodegenerative disorder characterized by memory loss, cognitive decline, and eventual impairment in daily functioning (Alzheimer's Disease Fact Sheet 2023). Psychotic symptoms occur in 10%–73% of patients with AD, though their underlying mechanisms remain incompletely understood. Evidence suggests a role of cholinergic dysfunction: reduced acetylcholinesterase activity and decreased non‐M2 receptor levels in the orbitofrontal cortex have been associated with psychosis in AD (Sinclair et al. 2019, Tsang et al. 2008). Current management is symptomatic, involving cholinesterase inhibitors, NMDA receptor antagonists, and anti‐amyloid immunotherapies (Smith et al. 2025, Matsunaga et al. 2015). However, no curative treatment exists.

Schizophrenia is a chronic psychiatric disorder marked by persistent delusions, hallucinations, disorganized thinking, and cognitive deficits, leading to substantial functional impairment (Schizophrenia). It has the highest global prevalence among neurological diseases and ranks among the top 20 causes of disability worldwide (GBD 2017 Disease and Injury Incidence and Prevalence Collaborators 2018). Dopamine D2 receptor–blocking antipsychotics remain the cornerstone of treatment, effectively controlling positive symptoms but often leaving negative and cognitive symptoms insufficiently addressed (Ceraso et al. 2020, Leber et al. 2024). Treatment‐resistant cases and adverse effects such as weight gain, extrapyramidal symptoms, and somnolence further complicate management (Howes et al. 2017, Kaul et al. 2024).

Alternative therapeutic strategies are under investigation. Pimavanserin, a selective 5‐HT2A receptor inverse agonist, demonstrated a significant reduction in psychosis among patients with probable AD psychosis without worsening cognition (Ballard et al. 2019, Ballard et al. 2018). Another promising candidate is KarXT, a dual‐acting muscarinic receptor modulator with both antipsychotic and precognitive effects, showing potential in both AD and schizophrenia (Brannan et al. 2021, Breier et al. 2023).

This review explores KarXT as a treatment option for psychosis in AD and schizophrenia, emphasizing its mechanism of action, efficacy, tolerability, and safety, highlighting how it may address limitations of traditional dopamine‐based therapies.

## 2 Methodology

A comprehensive literature search was conducted using four major databases—Google Scholar, PubMed, Scopus, and ClinicalTrials.gov. A total of 1162 records were identified (PubMed: 210; Scopus: 622; Google Scholar [first 30 pages]: 300; ClinicalTrials.gov: 30). After removal of 360 duplicate records, 802 unique records underwent primary screening. Of these, 702 records were excluded at the title/abstract level due to irrelevance to KarXT or non‐qualifying study types.

A total of 100 reports were selected for full‐text review, with none requiring retrieval from external sources. Following eligibility assessment, 61 full‐text articles were excluded (24 for using a different population, 19 for evaluating a different intervention, and 18 conference abstracts lacking complete data). Ultimately, 39 studies were included in the final narrative synthesis.

The inclusion criteria encompassed diverse study designs—preclinical investigations, clinical trials, and observational studies—focused on the efficacy, safety, mechanistic rationale, and broader therapeutic applications of KarXT in schizophrenia, AD psychosis, and other potential indications. Only English‐language studies were included to maintain clarity and consistency.

Search terms incorporated disease‐specific and pharmacological descriptors such as “adjunctive schizophrenia,” “AD‐related psychosis,” and “muscarinic acetylcholine receptor agonist.” The search included all available literature published up to February 2025.

**FIGURE 1:**
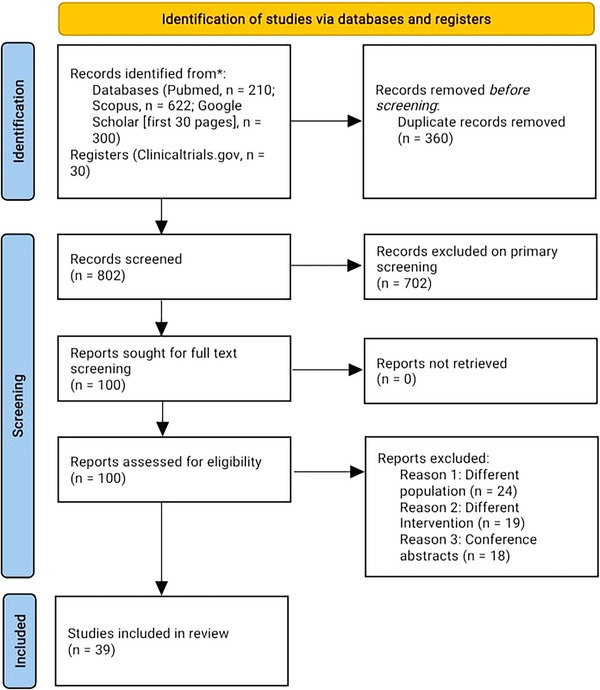
PRISMA flow diagram of study selection. The flowchart illustrates the identification, screening, eligibility assessment, and inclusion of studies in the narrative review.

The steps used in choosing a study are shown in Figure 1.

## 3 Unmet Needs in Schizophrenia and Psychosis in AD Management and Emerging Therapeutic Approaches

Around 47 million people were living with dementia in 2015, with the majority of them characterized as the most common form, i.e., AD (Livingston et al. 2017). More than half of the people with AD experience psychosis, delusions, and hallucinations (Murray et al. 2014). The estimated AD healthcare costs are approximately $305 billion in 2020 and are expected to rise to more than $1 trillion as the population grows (Wong 2020). Conversely, the estimated financial burden of schizophrenia in the U.S. alone doubled from 2013 to 2019. It held up at $343.2 billion in 2019 (Kadakia et al. 2022). These factors emphasize the importance of effective strategies and treatment options to effectively manage the difficult‐to‐treat patient population while ensuring its cost and availability to all socioeconomic groups.

The World Health Organization has recommended the use of aripiprazole, chlorpromazine, haloperidol, olanzapine, paliperidone, quetiapine, and risperidone as treatment options for adults with schizophrenia (Antipsychotic medicines for psychotic disorders). Clozapine should be considered for adults with a treatment‐resistant psychotic disorder (including schizophrenia) under mental health specialist supervision, carefully balancing effectiveness, side effects, and individual preference.

The first‐generation antipsychotic, Haloperidol, was associated with a significantly higher risk of extrapyramidal symptoms and Akathisia (Gao et al. 2008), and higher total hospitalization expenses (mean, U.S. $3215 per year) and total treatment cost (mean, $3769 per year) than olanzapine, zotepine, or quetiapine (Gau et al. 2008). From the limited available evidence, it is shown that chlorpromazine is also associated with a higher risk of patients developing extrapyramidal symptoms (Saha et al. 2016). The second‐generation antipsychotics, olanzapine, and risperidone were associated with the most significant weight gain, olanzapine also showed the largest BMI increase, and quetiapine XR showed a significant decrease in fasting glucose. Paliperidone showed the greatest increase in total cholesterol, though it also led to a positive rise in HDL levels. Aripiprazole reported the largest increase in triglycerides. Quetiapine reported the highest rise in systolic and diastolic pressures, but it was the only medication to report a decrease in fasting glucose (Rognoni et al. 2021).

First‐generation antipsychotics are a more costly option for the treatment of schizophrenia, especially haloperidol, as revealed by a study published in the bulletin of the WHO (Chisholm et al. 2008). They also come bearing some notable side effects, as mentioned earlier. Second‐generation antipsychotic medications, on the other hand, are associated with less severe extrapyramidal symptoms and better treatment outcomes than FGAs, that too only in the case of clozapine (Divac et al. 2014), they are also linked to greater chances of weight gain. An RCT evaluating the predictors of antipsychotic induced‐weight gain shows that patients treated with two SGAs, in particular, i.e., olanzapine (77%) and risperidone (63%), were more likely to experience weight gain when compared to haloperidol (22%) (Saddichha et al. 2008). All these limitations are a cause for concern. These higher costs not only make it harder for the privileged community to seek treatment but also make it nearly impossible for the treatment to reach the underserved community.

As far as therapeutic options for AD are concerned, AChEIs, donepezil, galantamine, rivastigmine, and the NMDA antagonist memantine are the only FDA‐approved AD medications (Yiannopoulou and Papageorgiou 2020). Rivastigmine is a “reversible” inhibitor of acetylcholinesterase and butyrylcholinesterase. It has been shown to improve cognition, but only when started as early as 6 months (Farlow et al. 2000). Donepezil, being a reversible inhibitor of AChE, only provides symptomatic relief and does not treat the underlying cause. Additionally, withdrawal of donepezil is associated with a higher probability of nursing home placement, which adds to the economic burden associated with AD (Howard et al. 2015). A meta‐analysis conducted by Andrea C et al. concluded that cognitive enhancers like memantine, in general, have minimal effects on cognition either in monotherapy or in conjunction with donepezil. These drugs appear safe, but must be used with caution in clinical practice, as trial participants might have less severe comorbidities (Tricco et al. 2018).

## 4 Introducing KarXT as a Novel Therapeutic Agent

Among the existing treatment options, one promising candidate is KarXT, introduced in late 2024 and marketed as COBENFY (FDA Approves Drug with New Mechanism of Action for Treatment of Schizophrenia | FDA). Ongoing clinical trials are investigating its treatment profile as an adjunctive therapy with standard‐of‐care agents for treating schizophrenia and alzheimer's dementia‐related psychosis. Data are expected in 2025 and 2026, respectively, as reported by Bristol Myers Squibb (Bms.com).

KarXT has a unique tolerability profile, with mild, transient side effects mostly occurring in the first 1–2 weeks, primarily related to pro‐cholinergic or anticholinergic events, and did not result in treatment discontinuations (Fabiano et al. 2024). This drug may address unmet needs in managing both Schizophrenia and AD‐related psychosis.

The EMERGENT‐3 trial evaluated KarXT in patients with schizophrenia experiencing acute psychosis over five weeks (Kaul et al. 2024). Xanomeline, an oral M1/M4‐selective muscarinic receptor agonist, does not interact with dopamine receptors, while trospium chloride, a highly polar anticholinergic agent, does not cross the blood‐brain barrier. The combination improves positive and negative symptoms (Leber et al. 2024, Staskin et al. 2010). The trial demonstrated an 8.4‐point greater reduction in PANSS total scores compared to placebo, without adverse effects such as extrapyramidal symptoms, weight gain, or sedation (Kaul et al. 2024). Side effects like tardive dyskinesia require longer follow‐up to establish safety.

The ADEPT‐1 phase 3 trial is a 38‐week, randomized, double‐blind, placebo‐controlled study in subjects with AD psychosis, assessing relapse prevention and safety/tolerability of KarXT (ClinicalTrials.gov ID: NCT05511363). ADEPT‐3 is a 52‐week open‐label extension for patients completing ADEPT‐1 or ADEPT‐2 (KarXT in Psychosis Associated With Alzheimer's Disease—Clinical Trials Registry—ICH GCP).

Given the burden of 6.7 million AD patients over 60 in the U.S. alone with psychosis, there is no FDA‐approved treatment (2023 Alzheimer's disease facts and figures 2023). KarXT's M1 receptor activity may enhance cognition (Bymaster et al. 2003), and dual M1/M4 action suggests it may be a potential treatment for AD psychosis as well as schizophrenia.

The FDA approves KarXT (COBENFY) for schizophrenia (https://www.accessdata.fda.gov/drugsatfda_docs/label/2024/216158s000lbl.pdf), with common side effects including nausea, vomiting, constipation, hypertension, tachycardia, and dizziness. Warnings exist for urinary retention, pre‐existing liver disease, and narrow‐angle glaucoma (https://www.accessdata.fda.gov/drugsatfda_docs/label/2024/216158s000lbl.pdf). Long‐term data from BMS suggest no metabolic changes or movement disorders in patients using KarXT for one year (Barry and Jewett 2024).

## 5 Mechanism of Action of KarXT

KarXT combines xanomeline (an M1/M4 muscarinic receptor agonist) and trospium chloride (a peripherally restricted muscarinic antagonist) to target CNS pathways while minimizing peripheral side effects, modulating cholinergic neurotransmission to reduce psychosis symptoms in schizophrenia and potentially AD (Kaul et al. 2024).

Preclinical and early‐phase clinical studies with selective M1 agonists such as HTL0018318 have demonstrated procognitive potential in AD and related populations (Nathan et al. 2022, Bakker et al. 2021). Although KarXT has not yet been tested in AD, its dual M1/M4 activity suggests it may confer both antipsychotic and procognitive benefits.

### 5.1 Critical Appraisal of Mechanistic Rationale for AD Psychosis

Psychosis in AD is multifactorial, involving cholinergic deficits, dopaminergic dysregulation, cortical‐subcortical network disruption, and neurodegenerative pathology such as amyloid plaques and neurofibrillary tangles. Cholinergic dysfunction–including loss of basal forebrain cholinergic neurons and reduced cortical acetylcholine–has been implicated in both cognitive decline and psychotic manifestations, providing a mechanistic basis for muscarinic receptor‐targeted therapies (Sinclair et al. 2019, Tsang et al. 2008). Xanomeline, as an M1/M4‐selective muscarinic receptor agonist, is hypothesized to restore cholinergic signaling, improve cognition, and reduce psychotic symptoms. Preclinical studies show that M1 activation enhances synaptic plasticity and modulates dopaminergic neurotransmission, potentially mitigating hallucinations and delusions (Nathan et al. 2022, Bakker et al. 2021). However, many contributing mechanisms of AD psychosis–such as amyloid and tau pathology, neuroinflammation, and network‐level disruptions–are not directly addressed by muscarinic modulation. Thus, while the mechanistic rationale for xanomeline is biologically plausible, it remains partially inferential, and clinical confirmation in Alzheimer's populations is essential to determine the extent to which muscarinic agonism alone can alleviate psychotic symptoms. This critical appraisal underscores the importance of ongoing trials to evaluate xanomeline's efficacy in the context of AD's complex and heterogeneous pathophysiology.

### 5.2 Overview of Xanomeline and Trospium Chloride Components

#### 5.2.1 Xanomeline

- Selective M1/M4 agonist.
- M1 receptors on neostriatum and neocortex projection neurons; activation increases dopamine release.
- M4 receptors on striatal cholinergic interneurons; activation reduces cAMP and regulates cholinergic output (Abrams et al. 2006; Shin et al. 2015; Yohn et al. 2022; Zhang et al. 2002; van der Westhuizen et al. 2021).
- Shows some selectivity at M1 over M2 receptors but equivalent efficacy at other subtypes, except M3, where all compounds are full agonists due to high receptor reserve (Mirza et al. 2003).
- Peripheral M1 activation historically caused gastrointestinal side effects (Bymaster et al. 2003; Singh 2022; Andersen et al. 2003).

#### 5.2.2 Trospium Chloride

- Peripherally restricted mAChR antagonist (M2/M3).
- Reduces undesired peripheral effects of xanomeline without crossing the BBB (Schmidt et al. 1994; Weiden et al. 2022).

This combination KarXT maximizes CNS M1/M4 activation while minimizing peripheral adverse events (Breier et al. 2023).

### 5.3 Mechanistic Rationale in Schizophrenia and AD

- Cholinergic dysregulation contributes to psychotic symptoms and cognitive deficits in schizophrenia (Mirza et al. 2003).
- AD patients experience degeneration of cholinergic neurons, exacerbating cognitive decline and psychosis (Sinclair et al. 2019).
- KarXT restores cholinergic signaling via selective M1/M4 activation, improving cognition and reducing psychosis without D2‐related extrapyramidal effects (Kaul et al. 2024).
- EMERGENT trials in schizophrenia and early AD studies support a transdiagnostic muscarinic mechanism [Bodick 1997].

### 5.4 Clinical Implications

- KarXT targets both negative and positive symptoms with minimal side effects (Kaul et al. 2024).
- Shows cognitive improvement in patients with impairment (Kaul et al. 2024).
- Provides a safer alternative for older adults with AD, who often poorly tolerate traditional antipsychotics (KarXT | ALZFORUM).

## 6 Pharmacological Profile of KarXT

### 6.1 Pharmacokinetics and Pharmacodynamics

#### 6.1.1 Pharmacokinetics

- Xanomeline: short half‐life, crosses blood brain barrier (BBB), low oral bioavailability, peak plasma in ∼2.5 h (Bender et al. 2017).
- Trospium: slow absorption, peripherally restricted, renal excretion, half‐life 10–20 h (Doroshyenko et al. 2005).

#### 6.1.2 Pharmacodynamics

- Xanomeline: dual M1/M4 agonist, no D2 activity (Kaul et al. 2024).
- KarXT reduces peripheral adverse events via Trospium (Fabiano et al. 2024).
- M1 muscarinic receptor → Gq protein → phospholipase C activation → increase in intracellular calcium (Ca^2^^+^)
- M4 muscarinic receptor → Gi protein → decrease in cyclic adenosine monophosphate (cAMP)

**FIGURE 2:**
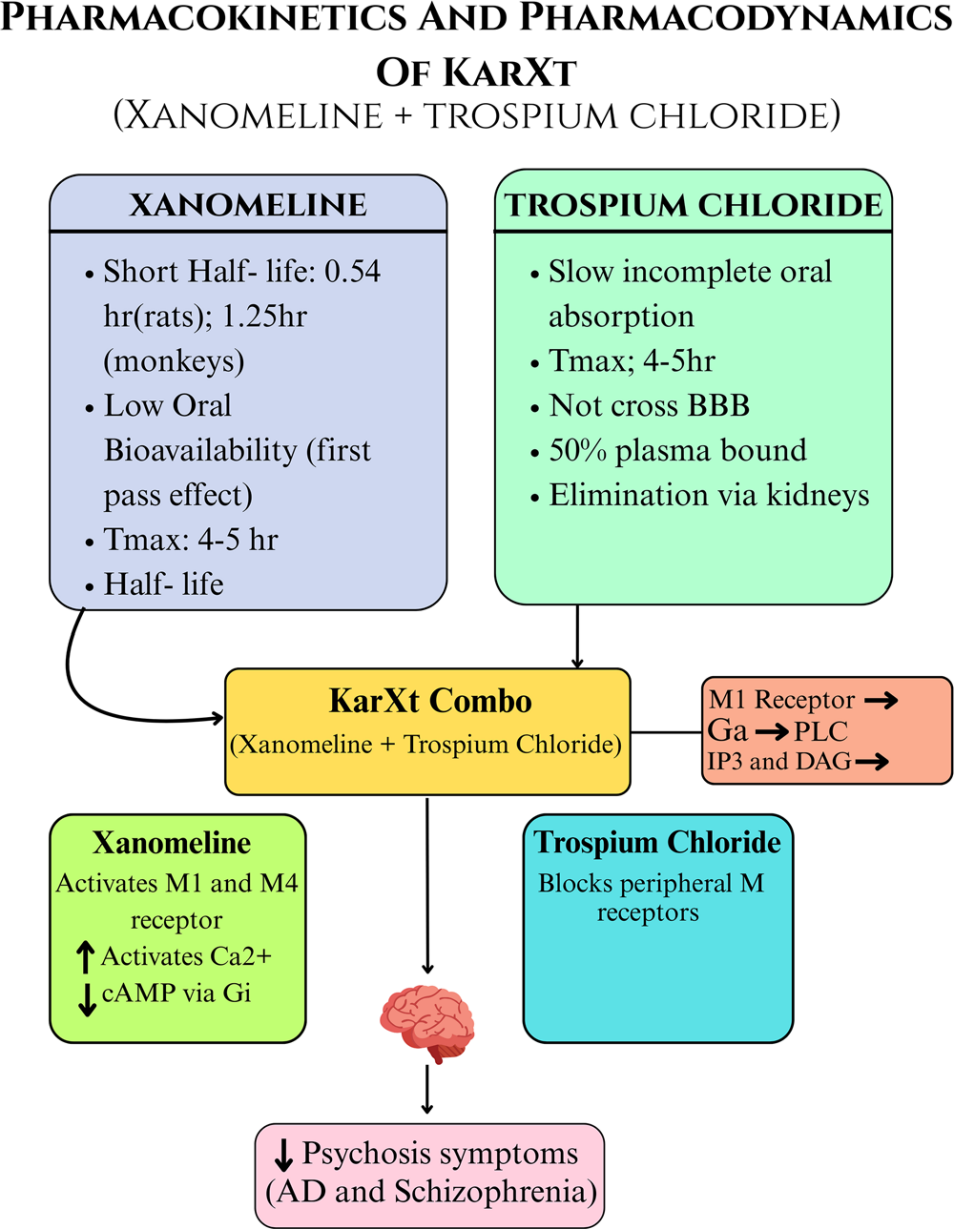
The image illustrates the pharmacokinetics and pharmacodynamics of KarXT, a combination of xanomeline and trospium chloride. Xanomeline's short half‐life and low oral bioavailability contrast with trospium chloride's slow absorption and inability to cross the blood‐brain barrier. KarXT's mechanism involves xanomeline activating M1 and M4 receptors and trospium chloride blocking peripheral muscarinic receptors, ultimately reducing psychosis symptoms.

A visual representation of both pharmacokinetics and pharmacodynamics is provided in Figure 2.

### 6.2 Dosing and Administration

- Schizophrenia: FDA‐approved, oral, starting 50 mg/20 mg BID, titrate to 100 mg/20 mg BID or max 125 mg/30 mg BID (FDA Approves Drug with New Mechanism of Action for Treatment of Schizophrenia | FDA, Kaul et al. 2024, Correll et al. 2022).
- AD: Phase 3 trials, flexible dosing 50 mg/20 mg BID, titrate to 100 mg/10 mg BID or max 200 mg/20 mg daily, depending on efficacy/tolerability ([Link], [Link], Bodick et al. 1997).

## 7 Clinical Efficacy of KarXT

Large‐scale clinical trials have put KarXT in a breakthrough position in treating schizophrenia and AD psychosis, for which it is still under trial with great success at targeting a wide range of symptoms, such as positive, negative, and cognitive symptoms of the disorder (Kaul et al. 2024, Kaul et al. 2024, [Link], Bristol Myers Squibb—Bristol Myers Squibb Presents New Interim Long‐Term Efficacy Data from the EMERGENT‐4 Trial Evaluating KarXT in Schizophrenia at the 2024 Annual Congress of the Schizophrenia International Research Society). The EMERGENT trials were built sequentially upon one another in the development of KarXT, starting from showing initial efficacy in EMERGENT‐1, acute settings in EMERGENT‐2, and confirming long‐term benefits and safety in EMERGENT‐3. The EMERGENT trials all followed the same study design: a phase 2 or phase 3, randomized, double‐blind, placebo‐controlled methodology, over a five‐week treatment period (Vuocolo et al. 2025).

EMERGENT‐1 was the first pivotal trial and laid the foundation for subsequent research, demonstrating significant reductions in both positive and negative symptoms of schizophrenia, with additional signals suggesting improvement in cognitive functioning (Correll et al. 2022).

Based on these results, EMERGENT‐2, a phase 3 trial, evaluated the safety and efficacy of KarXT in a larger cohort of patients with acute psychosis. Conducted from December 2020 to April 2022, the trial enrolled 252 participants (aged 18–65) across 22 sites in the USA. KarXT achieved the primary endpoint, with a mean change from baseline to week 5 in the positive and negative syndrome scale (PANSS) total score (–21.2 points) versus placebo (–11.6 points), and secondary endpoints—including cognitive function and overall safety—were also met (*p* < 0.05) (Kaul et al. 2024).

The next EMERGENT‐3 trial, also a phase 3 study, built upon these findings and focused on the long‐term efficacy and safety of KarXT for schizophrenia, particularly symptom recurrence and sustained improvement. The results verified that KarXT continues to afford significant improvement of symptoms for a considerable duration, encompassing acute and chronic aspects of schizophrenia (Kaul et al. 2024).

Additionally, Bristol Myers Squibb pre‐released findings from the EMERGENT‐4 trial regarding the long‐term safety and effectiveness of KarXT (currently Cobenfy) over 52 weeks for those who had previously participated in the EMERGENT trials. More than 75% of participants recorded >30% improvement from baseline on the PANSS total score, confirming its efficacy over longer durations (Bristol Myers Squibb—Bristol Myers Squibb Presents New Interim Long‐Term Efficacy Data from the EMERGENT‐4 Trial Evaluating KarXT in Schizophrenia at the 2024 Annual Congress of the Schizophrenia International Research Society). Based on these results, Cobenfy was approved by the FDA in September 2024 for oral administration in the treatment of schizophrenia in adult patients (FDA Approves Drug with New Mechanism of Action for Treatment of Schizophrenia | FDA, Kingwell 2024, Dolgin 2024).

Furthermore, KarXT is being studied for its potential to treat psychosis related to AD. The ADEPT‐2 trial is an interventional clinical study assessing the safety and efficacy of KarXT in adults aged 55–90 years with mild to severe AD–related psychosis ([Link], [Link]). The primary objective is to determine the extent of reduction in hallucinations and delusions (A Study to Assess Efficacy and Safety of KarXT for the Treatment of Psychosis Associated With Alzheimer's Disease (ADEPT‐2)). Results from ADEPT‐2 and ADEPT‐4 are pending, with trial completion dates in July 2025 and October 26, 2025, respectively (A Study to Evaluate KarXT as a Treatment for Psychosis Associated With Alzheimer's Disease (ADEPT‐4)). Nevertheless, the innovative muscarinic‐receptor–targeted mechanism of KarXT remains promising for addressing both cognitive and psychotic symptoms in AD (Therapeutic Areas | BMS Science | HCP Site).

In summary, KarXT has yielded encouraging outcomes in clinical trials of schizophrenia and shows potential for treating AD psychosis as well. It has greatly improved psychotic symptoms and provides a novel therapeutic mechanism that overcomes the shortcomings of standard antipsychotic drugs (Brannan et al. 2021) (Huhn et al. 2020). Table 1 presents the summary of these trials [(Kaul et al. 2024, Kaul et al. 2024, [Link], Bristol Myers Squibb—Bristol Myers Squibb Presents New Interim Long‐Term Efficacy Data from the EMERGENT‐4 Trial Evaluating KarXT in Schizophrenia at the [Link], [Link], A Study to Evaluate KarXT as a Treatment for Psychosis Associated With Alzheimer's Disease (ADEPT‐4))].

**TABLE 1:** Summary of study designs and results: This table outlines current available trials KarXT for the schizophrenia and psychosis in alzheimer's disease, detailing their design, participant demographics, gender distribution, study duration, adverse events of KarXT and key findings with author's conclusion.

Study name	Design	Study location	Participants	Gender	Duration	Results	TEAEs	Authors conclusion
EMERGENT TRIAL‐1 (Karuna therapeutics) (Study Details | A Study to Assess Safety and Efficacy of KarXT in Adult Patients With Schizophrenia | ClinicalTrials.gov)	Randomized, double‐blind, placebo‐controlled, phase 2 study	Multi‐center study involving multiple clinical sites.	Adults aged 18 to 60 years	Both male and female participants. The study did not exclude participants based on gender	Five weeks	Reduction in the positive and negative syndrome scale (PANSS) total score compared to placebo at week 5, with an effect size of 0.75 (*p* < 0.0001)	Common adverse events associated with KarXT included constipation, nausea, dry mouth, dyspepsia, and vomiting; these were mostly mild to moderate and did not lead to treatment discontinuation	Concluded that the combination of xanomeline and trospium (KarXT) was superior to placebo in reducing the total score on the positive and negative syndrome scale (PANSS) in adult patients with schizophrenia.
EMERGENT TRIAL 2 (Karuna therapeutics) (Kaul et al. 2024)	Phase 3, randomized, double‐blind, placebo‐controlled, flexible‐dose	Conducted across 22 inpatient sites in the USA, including locations in Florida, Georgia, Illinois, Missouri, Nevada, New Jersey, Ohio, and Texas.	Adults aged 18 to 65 years with a diagnosis of schizophrenia.	Both male and female participants.	December 2020 to April 2022.	Change from baseline to week 5 in PANSS total score. Secondary outcome included the proportion of participants achieving at least a 30% reduction in PANSS total score.	(KarXT) was generally well‐tolerated by participants. There were no significant reports of weight gain or metabolic disturbances. Constipation, nausea, dry mouth, dyspepsia, and vomiting.	Xanomeline‐trospium (KarXT) demonstrated a statistically significant and clinically meaningful reduction in both positive and negative symptoms of schizophrenia compared to placebo. The trial met its primary endpoint, with KarXT showing a 9.6‐point greater reduction in the positive and negative Syndrome Scale (PANSS) total score than placebo at week 5 (*p* < 0.0001).
EMERGENT TRIAL 3 (Karuna therapeutics) (Kaul et al. 2024)	Phase 3, multicenter, randomized, double‐blind, placebo‐controlled study.	Conducted at 30 inpatient sites, comprising 18 locations in the United States and 12 in Ukraine.	Adults aged 18 to 65 years with a diagnosis of schizophrenia	Both male and female participants.	April 1, 2021, and December 7, 2022.	Primary outcome measure: change from baseline to week 5 in the positive and negative syndrome scale (PANSS) total score. Changes from baseline to week 5 in PANSS positive subscale score, PANSS negative subscale score, PANSS marder negative factor score, and clinical global impression‐severity (CGI‐S) score.	Generally well‐tolerated. Common treatment‐emergent adverse events (TEAEs) reported in the KarXT group were: Nausea: 19.2%, dyspepsia: 16.0%, vomiting: 16.0%, constipation: 12.8%	Concluded that xanomeline‐trospium was efficacious and well tolerated in individuals with schizophrenia experiencing acute psychosis. These findings, consistent with the results from the prior EMERGENT‐1 and EMERGENT‐2 trials.
EMERGENT TRIAL 4 (Bristol Myers Squibb—Bristol Myers Squibb Presents New Interim Long‐Term Efficacy Data from the EMERGENT‐4 Trial Evaluating KarXT in Schizophrenia at the 2024 Annual Congress of the Schizophrenia International Research Society)	Phase 3, 52‐week, open‐label extension study designed to evaluate the long‐term safety.	Trial was conducted at multiple outpatient sites.	156 participants aged 18 to 65 years	Both male and female participants were included.	52 weeks	Interim results demonstrated significant improvements in schizophrenia symptoms, with an average reduction of 33.3 points in PANSS total score from baseline at week 52. Additionally, over 75% of participants achieved a greater than 30% improvement in symptoms.	KarXT was not linked to extrapyramidal symptoms or significant changes in prolactin levels. Reported in at least 5% of participants. constipation, nausea, dry mouth, dyspepsia, and vomiting. These adverse events were predominantly mild to moderate in severity and transient in nature.	Concluded that long‐term treatment with Xanomeline‐trospium (KarXT) was safe and generally well tolerated in participants with schizophrenia. They observed significant improvements in schizophrenia symptoms, with more than 75% of participants achieving over a 30% reduction in symptoms, and an average reduction of 33.3 points from baseline in the positive and negative syndrome scale (PANSS) total score.
ADEPT‐ 2 Trial (A Study to Assess Efficacy and Safety of KarXT for the Treatment of Psychosis Associated With Alzheimer's Disease (ADEPT‐2))	Phase 3, randomized, double‐blind, placebo‐controlled, parallel‐group study.	The trial is actively recruiting across multiple countries, including Greece, Poland, and Turkey.	Male and female subjects aged 55 to 90 years.	Make and female both.	14 weeks.	Results for adept 2 trial are still pending.	Specific TEAE data for ADEPT‐2 are not yet publicly available.	As of now, the ADEPT‐2 trial, a phase 3 study evaluating KarXT xanomeline‐trospium (KarXT) for treating psychosis in alzheimer's disease patients, is ongoing, and the authors have not yet published their conclusions.
ADEPT 4 Trial (A Study to Evaluate KarXT as a Treatment for Psychosis Associated With Alzheimer's Disease (ADEPT‐4))	Phase 3, randomized, quadruple‐blind, placebo‐controlled study.	This trial is actively recruiting participants across multiple locations.	Adults aged 55 to 90 years.	Both male and female.	While the exact duration of the ADEPT‐4 trial is not specified in the available sources.	Results for ADEPT‐4 trial are still pending.	Specific TEAE data for ADEPT‐2 are not yet publicly available.	As of now, the ADEPT‐4 trial, a Phase 3 study evaluating xanomeline‐trospium (KarXT) for treating psychosis in alzheimer's disease patients, is ongoing, and the authors have not yet published their conclusions

Abbreviations: PANSS, positive and negative syndrome scale; CGI‐S, clinical global impression‐severity; ADEPT, Adaptive Implementation of Effective Programs Trial; NIH, National Institute of Health; TEAE, treatment emergent adverse events; USA, United States of America.

## 8 Safety and Tolerability Profile

KarXT has demonstrated a favorable safety and tolerability profile across the EMERGENT trials, with most adverse events being mild to moderate and transient. The most common treatment‐emergent adverse events reported in these trials included gastrointestinal disturbances such as nausea, constipation, and vomiting, as well as mild, transient increases in blood pressure (Kaul et al. 2024, Dolgin 2024). All pro‐cholinergic adverse events were short‐lived. Long‐term monotherapy showed high patient compliance and no significant changes in body weight, metabolic parameters, somnolence, or vital signs typically associated with conventional antipsychotic medications (Bristol Myers Squibb—Bristol Myers Squibb Presents New Interim Long‐Term Efficacy Data from the EMERGENT‐4 Trial Evaluating KarXT in Schizophrenia at the 2024 Annual Congress of the Schizophrenia International Research Society, Huhn et al. 2020, Kramer et al. 2025). While safety data in elderly populations are increasingly available, information on other at‐risk groups, including pregnant or lactating women and patients with comorbid conditions, remains limited, highlighting areas for future investigation.

## 9 Potential Advantages of KarXT

**FIGURE 3:**
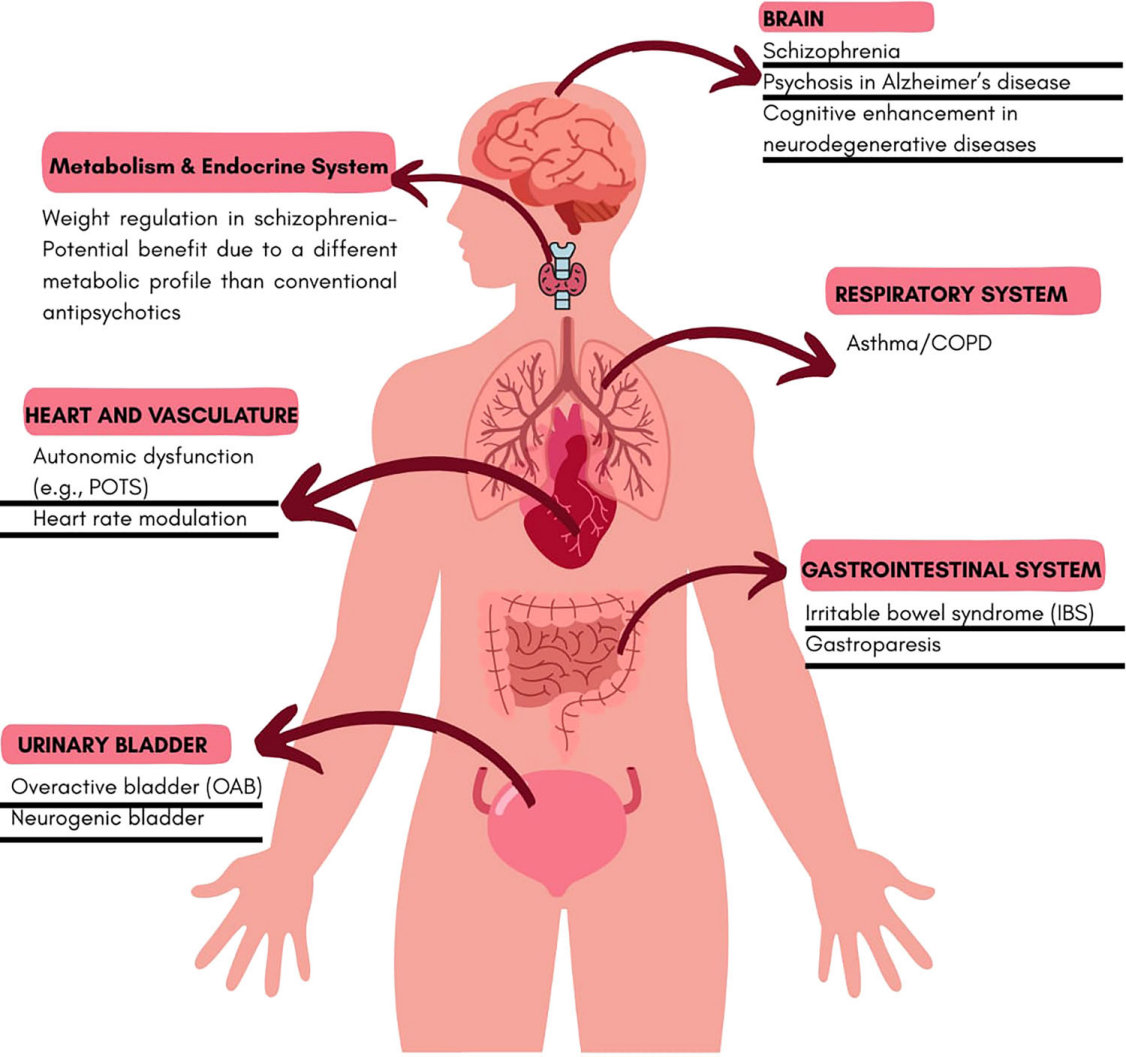
The image depicts the diverse therapeutic potential of KarXT across various organ systems. Beyond its psychiatric uses in schizophrenia and Alzheimer's psychosis, it suggests benefits in respiratory, gastrointestinal, urological, cardiovascular, and metabolic conditions, indicating a broad range of applications.

Based on trial findings, KarXT exhibits a favorable metabolic profile, minimizing weight gain and the risk of metabolic syndrome commonly observed with dopamine‐based antipsychotics (Azargoonjahromi 2024, Serafini et al. 2016). Clinical trials indicate that KarXT does not significantly elevate prolactin levels, reducing the likelihood of endocrine‐related side effects such as menstrual disturbances and osteoporosis (Much Ado About Something: Why There's Excitement Behind Novel Mechanism of Action in Schizophrenia). Cardiovascular effects reported in trials were generally mild and transient, with only minor increases in heart rate or blood pressure in some patients (KarXT | ALZFORUM). In the urinary system, KarXT may alleviate symptoms of overactive bladder, although there remains a theoretical risk of urinary retention ([Link], [Link]). Respiratory effects observed in trials were minimal (Azargoonjahromi 2024,). Overall, KarXT's selective muscarinic action presents a promising alternative with reduced metabolic and endocrine burden; however, long‐term effects across multiple organ systems have yet to be fully established (Vasiliu et al. 2024). A visual representation is provided in Figure 3.

## 10 Limitations

This study primarily explores treatment options for psychosis in both schizophrenia and AD, and several limitations should be acknowledged. First, data for alzheimer's‐related psychosis remain limited compared with schizophrenia. Second, the focus of this review is symptomatic management rather than addressing underlying disease mechanisms, such as amyloid plaque accumulation and neurofibrillary tangles in AD. While symptomatic management is important, it may not substantially improve long‐term quality of life, underscoring the need for therapies targeting disease progression. Third, treatment responses observed in clinical trials may differ from real‐world outcomes, as trials are time‐limited and often exclude patients with complex comorbidities. Fourth, potential drug interactions may not yet be fully identified.

Additionally, as a narrative review, this study has inherent limitations: it does not include a formal quality appraisal or risk‐of‐bias assessment of the included studies; it may be prone to selection and publication bias; the exclusion of non‐English publications could have limited the comprehensiveness of the evidence; and study selection and interpretation are influenced by the authors’ subjective judgment. These aspects should be considered when interpreting the findings, and future systematic reviews with rigorous methodology are warranted to validate and expand upon these conclusions.

## 11 Future Directions in Research and Clinical Practice

Ongoing clinical trials suggest that KarXT has the potential to provide meaningful symptom relief in schizophrenia and AD psychosis while mitigating the side effects associated with xanomeline monotherapy (UW Clinical Trials Institute | New schizophrenia drug could treat Alzheimer's disease). Further research remains crucial to confirm the efficacy, safety, and tolerability of KarXT across diverse populations (Azargoonjahromi 2024). The trials have highlighted the occurrence of pro‐cholinergic and anticholinergic adverse events, which are linked to the drug's mechanism of action. These events were more frequent in the KarXT group compared with placebo, but none were classified as severe, and no participants discontinued treatment due to adverse events, indicating that they were generally manageable (Kaul et al. 2024, Correll et al. 2022). Most adverse events appeared within the first week and were transient, with durations ranging from 1 day for vomiting to 13 days for dry mouth. Similar events were also observed in placebo groups (Correll et al. 2022). These findings suggest that KarXT's tolerability may support better adherence and potentially improve long‐term outcomes compared with current antipsychotics (Correll et al. 2022).

KarXT demonstrated broad improvements across multiple symptom domains early in treatment, supporting its potential as a novel antipsychotic based on muscarinic receptor agonism (Weiden et al. 2022). Its combination of xanomeline and trospium chloride enhances central cholinergic signaling while minimizing peripheral effects, making it a promising therapeutic option. Regulatory approval by the U.S. food and drug administration (FDA) for oral use in adults with schizophrenia in September 2024 further emphasizes the importance of synthesizing evidence on its safety, tolerability, and clinical benefits (Fabiano et al. 2024).

Considering the encouraging findings in schizophrenia, KarXT is now being evaluated in Phase III trials for psychosis associated with AD (Kaul et al. 2024). Preliminary observations indicate a broad spectrum of benefits in alzheimer's patients, including reductions in hallucinations, delusions, hostility, and vocal outbursts (Psychosis: The Other Big Alzheimer's Therapeutic Target—BioSpace). Data from the EMERGENT‐4 and EMERGENT‐5 studies confirm that KarXT is generally well‐tolerated, with minimal weight gain or metabolic issues, and some obese participants even experienced weight loss (Evaluating Pooled Data from the EMERGENT Study Programs). Continued investigation will be essential to determine long‐term safety, cognitive effects, and real‐world effectiveness, potentially establishing KarXT as a new class of antipsychotic therapy.

## 12 Conclusion

Psychosis is a common feature of schizophrenia and AD, most often caused by high concentrations of the neurotransmitter dopamine in the brain. It is characterized by hallucinations, delusions, and disorganized thinking, resulting in a markedly reduced quality of life for the patient and associated caregiver burden. Conventional treatments target dopamine receptors as their mechanism of action, which results in undesirable side effects like extrapyramidal symptoms, limiting their use and increasing the chances of patients' noncompliance with medication. A large number of treatment options have been studied. However, this article explores a newly FDA‐approved drug, KarXT. KarXT, with its dual action on the M1 and M4 receptors and mAChR antagonism, greatly helps reduce the severity of the positive and negative symptoms, as is evident by the PANSS scale. It resulted in an 8.4‐point greater reduction associated with schizophrenia and AD. The side effects experienced were minimal and did not account for the discontinuation of treatment. KarXT has demonstrated encouraging results across various clinical trials, including the ADEPT and EMERGENT phase 2 and phase 3 studies. These trials highlighted its robust safety profile, excellent tolerability, and notable efficacy in the improvement of cognitive symptoms. This dual benefit positions KarXT as a promising treatment option for treating schizophrenia and AD. The positive results observed in these trials offer significant hope for patients, suggesting that KarXT may play a vital role in the management of these disorders. The FDA has approved the use of KarXT for acute psychosis associated with Schizophrenia as an oral formulation, but it is still under trial for the condition of AD. It has a short half‐life and is well excreted by the kidneys. Therefore, dose adjustment is required in renally impaired patients.

## Author Contributions

Ashir Kanwal had the idea of the project. Literature search was performed by Ashir Kanwal, Bismah Azeem, Fatima Mumtaz and Hania Nasir. Draft of the manuscript was written by Ashir Kanwal, Bismah Azeem, Hania Nasir and Fatima Mumtaz. Ashir Kanwal and Mohammed Hammad Jaber Amin aligned the manuscript. The draft was reviewed and edited by Bilal Wazir Khan, Nauman Ashraf and Mohammed Hammad Jaber Amin. Warisha Kanwal constructed a graphical abstract for the manuscript. All the authors agreed to be accountable for all aspects of the work in ensuring that questions related to the accuracy or integrity of any part of the work are appropriately investigated and resolved.

## Funding

The authors have nothing to report.

## Ethics Statement

The authors have nothing to report.

## Consent

The authors have nothing to report.

## Conflicts of Interest

The authors declare no conflicts of interest.

## Data Availability

The data used to support the findings of this study are included within the article.

## References

[brb371182-bib-0001] 2023 Alzheimer's disease facts and figures . 2023. “Alzheimer's and Dementia.” The journal of the Alzheimer's Association 19, no. 4: 1598–1695. 10.1002/alz.13016. 36918389

[brb371182-bib-0002] “A Study to Assess Efficacy and Safety of KarXT for the Treatment of Psychosis Associated With Alzheimer's Disease (ADEPT‐2) .” Accessed: Feb. 23, 2025. [Online]. Available: https://ctv.veeva.com/study/a‐study‐to‐assess‐efficacy‐and‐safety‐of‐karxt‐for‐the‐treatment‐of‐psychosis‐associated‐with‐alzhei‐1.

[brb371182-bib-0003] “A Study to Evaluate KarXT as a Treatment for Psychosis Associated With Alzheimer's Disease (ADEPT‐4) .” Accessed: Feb. 23, 2025. [Online]. Available: https://ctv.veeva.com/study/a‐study‐to‐evaluate‐karxt‐as‐a‐treatment‐for‐psychosis‐associated‐with‐alzheimers‐disease.

[brb371182-bib-0004] Abrams, P. , K. Andersson , J. J. Buccafusco , et al. 2006. “Muscarinic Receptors: Their Distribution and Function in Body Systems, and the Implications for Treating Overactive Bladder.” British Journal of Pharmacology 148, no. 5: 565–578. 10.1038/sj.bjp.0706780. 16751797 PMC1751864

[brb371182-bib-0005] “Alzheimer's Disease Fact Sheet.” National Institute on Aging, National Institutes of Health 2023. www.nia.nih.gov/health/alzheimers‐and‐dementia/alzheimers‐disease‐fact‐sheet.

[brb371182-bib-0006] American Psychiatric Association Task Force on DSM‐IV . 2000. Diagnostic and Statistical Manual of Mental Disorders, 4th edition text revision (DSM‐IV‐TR). Washington, DC: American Psychiatric Association. https://img3.reoveme.com/m/2ab8dabd068b16a5.pdf.

[brb371182-bib-0007] Andersen, M. B. , A. Fink‐Jensen , L. Peacock , et al. 2003. “The Muscarinic M1/M4 Receptor Agonist Xanomeline Exhibits Antipsychotic‐Like Activity in Cebus Apella Monkeys.” Neuropsychopharmacology 28: 1168–1175. 10.1038/sj.npp.1300151. 12700711

[brb371182-bib-0008] Antipsychotic medicines for psychotic disorders . https://www.who.int/teams/mental‐health‐and‐substance‐use/treatment‐care/mental‐health‐gap‐action‐programme/evidence‐centre/psychosis‐and‐bipolar‐disorders/antipsychotic‐medicines‐for‐psychotic‐disorders#:~:text=Oral%20antipsychotic%20medicines%20%E2%80%93%20namely%20aripiprazole,side%2Deffects%20and%20individual%20preference.

[brb371182-bib-0009] Arciniegas, D. B. 2015. “Psychosis.” Behavioral Neurology and Neuropsychiatry 21, no. 3: 715–736. 10.1212/01.CON.0000466662.89908.e7. PMC4455840 26039850

[brb371182-bib-0010] Azargoonjahromi, A. 2024. “Current Findings and Potential Mechanisms of KarXT (Xanomeline‐Trospium) in Schizophrenia Treatment.” Clinical Drug Investigation 44, no. 7: 471–493. 10.1007/S40261-024-01377-9. 38904739

[brb371182-bib-0011] Bakker, C. , T. Tasker , J. Liptrot , et al. 2021. “Safety, Pharmacokinetics and Exploratory Pro‐Cognitive Effects of HTL0018318, a Selective M1 Receptor Agonist, in Healthy Younger Adult and Elderly Subjects: A Multiple Ascending Dose Study.” Alzheimer's researchand therapy 13, no. 1: 87. 10.1186/s13195-021-00816-5. PMC8061066 33883008

[brb371182-bib-0012] Ballard, C. , C. Banister , Z. Khan , et al. 2018. “Evaluation of the Safety, Tolerability, and Efficacy of pimavanserin Versus Placebo in Patients With Alzheimer's Disease Psychosis: A Phase 2, Randomised, Placebo‐Controlled, Double‐Blind Study.” The Lancet. Neurology 17, no. 3: 213–222. 10.1016/S1474-4422(18)30039-5. 29452684

[brb371182-bib-0013] Ballard, C. , J. M. Youakim , B. Coate , and S. Stankovic . 2019. “Pimavanserin in Alzheimer's Disease Psychosis: Efficacy in Patients With More Pronounced Psychotic Symptoms.” The journal of prevention of Alzheimer's disease 6, no. 1: 27–33. 10.14283/jpad.2018.30. PMC12280763 30569083

[brb371182-bib-0014] Barry, E. , and C Jewett . 2024. “F.D.A. approves the First New Schizophrenia Drug in Decades.” The New York times [Internet]. [cited 2025 Feb 5]; Available from:. https://www.nytimes.com/2024/09/26/health/fda‐schizophrenia‐drug.html.

[brb371182-bib-0015] Bender, A. M. , C. K. Jones , and C. W. Lindsley . 2017. “Classics in Chemical Neuroscience: Xanomeline.” ACS Chemical Neuroscience 8, no. 3: 435–443. 10.1021/acschemneuro.7b00001. 28141924

[brb371182-bib-0016] Bms.com . [cited 2025 Feb 5]. Available from:. https://news.bms.com/news/details/2023/Bristol‐Myers‐Squibb‐Strengthens‐Neuroscience‐Portfolio‐with‐Acquisition‐of‐Karuna‐Therapeutics/default.aspx.

[brb371182-bib-0017] Bodick, N. C. , W. W. Offen , A. I. Levey , et al. 1997. “Effects of Xanomeline, a Selective Muscarinic Receptor Agonist, on Cognitive Function and Behavioral Symptoms in Alzheimer disease.” Archives of neurology 54, no. 4: 465–473. 10.1001/archneur.1997.00550160091022. 9109749

[brb371182-bib-0018] Brannan, S. K. , S. Sawchak , A. C. Miller , J. A. Lieberman , S. M. Paul , and A. Breier . 2021. “Muscarinic Cholinergic Receptor Agonist and Peripheral Antagonist for Schizophrenia.” The New England journal of medicine 384, no. 8: 717–726. 10.1056/NEJMoa2017015. 33626254 PMC7610870

[brb371182-bib-0019] Breier, A. , S. K. Brannan , S. M. Paul , and A. C. Miller . 2023. “Evidence of trospium's Ability to Mitigate Cholinergic Adverse Events Related to Xanomeline: Phase 1 Study Results.” Psychopharmacology 240, no. 5: 1191–1198. 10.1007/s00213-023-06362-2. 37036495 PMC10102054

[brb371182-bib-0020] “Bristol Myers Squibb—Bristol Myers Squibb Presents New Interim Long‐Term Efficacy Data from the EMERGENT‐4 Trial Evaluating KarXT in Schizophrenia at the 2024 Annual Congress of the Schizophrenia International Research Society .” Accessed: Feb. 22, 2025. [Online]. Available: https://news.bms.com/news/details/2024/Bristol‐Myers‐Squibb‐Presents‐New‐Interim‐Long‐Term‐Efficacy‐Data‐from‐the‐EMERGENT‐4‐Trial‐Evaluating‐KarXT‐in‐Schizophrenia‐at‐the‐2024‐Annual‐Congress‐of‐the‐Schizophrenia‐International‐Research‐Society/default.aspx?utm_source=chatgpt.com.

[brb371182-bib-0021] Bymaster, F. P. , P. A. Carter , M. Yamada , et al. 2003. “Role of Specific Muscarinic Receptor Subtypes in Cholinergic Parasympathomimetic Responses, in Vivo Phosphoinositide Hydrolysis, and Pilocarpine‐Induced Seizure Activity.” The European journal of neuroscience 17, no. 7: 1403–1410. 10.1046/j.1460-9568.2003.02588.x. 12713643

[brb371182-bib-0022] Ceraso, A. , J. J. Lin , J. Schneider‐Thoma , et al. 2020. “Maintenance Treatment With Antipsychotic Drugs for Schizophrenia.” The Cochrane database of systematic reviews 8, no. 8: CD008016. 10.1002/14651858.CD008016.pub36. 32840872 PMC9702459

[brb371182-bib-0023] Chisholm, D. , O. Gureje , S. Saldivia , et al. 2008. “Schizophrenia Treatment in the Developing World: An Interregional and Multinational Cost‐Effectiveness Analysis.” Bulletin of the World Health Organization 86, no. 7: 542–551. 10.2471/blt.07.045377. 18670667 PMC2647485

[brb371182-bib-0024] ClinicalTrials.gov ID: NCT05511363 . https://clinicaltrials.gov/study/NCT05511363.

[brb371182-bib-0025] “Cobenfy: A New Antipsychotic | CARLAT PUBLISHING .” Accessed: Mar. 20, 2025. [Online]. Available: https://www.thecarlatreport.com/blogs/2‐the‐carlat‐psychiatry‐podcast/post/4842‐cobenfy‐a‐new‐antipsychotic?utm_source=chatgpt.com.

[brb371182-bib-0026] Correll, C. U. , A. S. Angelov , A. C. Miller , P. J. Weiden , and S. K. Brannan . 2022. “Safety and Tolerability of KarXT (Xanomeline‐Trospium) in a Phase 2, Randomized, Double‐Blind, Placebo‐Controlled Study in Patients With Schizophrenia.” Schizophrenia (Heidelberg, Germany) 8, no. 1: 109. 10.1038/s41537-022-00320-1. 36463237 PMC9719488

[brb371182-bib-0027] Divac, N. , M. Prostran , I. Jakovcevski , and N. Cerovac . 2014. “Second‐Generation Antipsychotics and Extrapyramidal Adverse Effects.” BioMed research international 2014: 656370. 10.1155/2014/656370. 24995318 PMC4065707

[brb371182-bib-0028] Dolgin, E. 2024. “Revolutionary Drug for Schizophrenia Wins US Approval.” Nature 634, no. 8033: 276–277. 10.1038/D41586-024-03123-9. 39333421

[brb371182-bib-0029] Doroshyenko, O. , A. Jetter , K. P. Odenthal et al. 2005. “Clinical Pharmacokinetics of Trospium Chloride.” Clinical Pharmacokinetics 44: 701–720. 10.2165/00003088-200544070-00003. 15966754

[brb371182-bib-0030] “Evaluating Pooled Data from the EMERGENT Study Programs .” Accessed: Jan. 31, 2025. [Online]. Available:. https://www.psychiatrist.com/news/evaluating‐pooled‐data‐from‐the‐emergent‐study‐programs/.

[brb371182-bib-0031] Fabiano, N. , S. Wong , C. Zhou , C. U. Correll , M. Højlund , and M. Solmi . 2024. “Efficacy, Tolerability, and Safety of Xanomeline‐trospium chloride for Schizophrenia: A Systematic Review and Meta‐Analysis.” European neuropsychopharmacology: The journal of the European College of Neuropsychopharmacology 92: 62–73. 10.1016/j.euroneuro.2024.11.013. 39724748

[brb371182-bib-0032] Farlow, M. , R. Anand , J. Messina Jr , R. Hartman , and J Veach,. 2000. “A 52‐Week Study of the Efficacy of Rivastigmine in Patients With Mild to Moderately Severe Alzheimer's Disease.” European neurology 44, no. 4: 236–241. 10.1159/000008243. 11096224

[brb371182-bib-0033] “FDA Approves Drug with New Mechanism of Action for Treatment of Schizophrenia | FDA .” Accessed: Feb. 23, 2025. [Online]. Available: https://www.fda.gov/news‐events/press‐announcements/fda‐approves‐drug‐new‐mechanism‐action‐treatment‐schizophrenia.

[brb371182-bib-0034] Gao, K. , D. E. Kemp , S. J. Ganocy , P. Gajwani , G. Xia , and J. R. Calabrese . 2008. “Antipsychotic‐induced Extrapyramidal Side Effects in Bipolar Disorder and Schizophrenia: A Systematic Review.” Journal of clinical psychopharmacology 28, no. 2: 203–209. 10.1097/JCP.0b013e318166c4d5. 18344731 PMC3489178

[brb371182-bib-0035] Gau, S. S. , C. H. Chung , and C. S. Gau . 2008. “A Pharmacoeconomic Analysis of Atypical Antipsychotics and Haloperidol in First‐Episode Schizophrenic Patients in Taiwan.” Journal of clinical psychopharmacology 28, no. 3: 271–278. 10.1097/JCP.0b013e318172371313. 18480683

[brb371182-bib-0036] GBD 2017 Disease and Injury Incidence and Prevalence Collaborators . 2018. “Global, Regional, and National Incidence, Prevalence, and Years Lived With Disability for 354 Diseases and Injuries for 195 Countries and territories, 1990‐2017: A Systematic Analysis for the Global Burden of Disease Study 2017.” Lancet (London, England) 392, no. 10159: 1789–1858. 10.1016/S0140-6736(18)32279-74. 30496104 PMC6227754

[brb371182-bib-0037] Howard, R. , R. McShane , J. Lindesay , et al. 2015. “Nursing Home Placement in the Donepezil and Memantine in Moderate to Severe Alzheimer's Disease (DOMINO‐AD) Trial: Secondary and Post‐Hoc Analyses.” The Lancet. Neurology 14, no. 12: 1171–1181. 10.1016/S1474-4422(15)00258-6. 26515660

[brb371182-bib-0038] Howes, O. D. , R. McCutcheon , O. Agid , et al. 2017. “Treatment‐Resistant Schizophrenia: Treatment Response and Resistance in Psychosis (TRRIP) Working Group Consensus Guidelines on Diagnosis and Terminology.” The American Journal of Psychiatry 174, no. 3: 216–229. 10.1176/appi.ajp.2016.16050503. 27919182 PMC6231547

[brb371182-bib-0039] https://www.accessdata.fda.gov/drugsatfda_docs/label/2024/216158s000lbl.pdf.

[brb371182-bib-0040] https://www.accessdata.fda.gov/drugsatfda_docs/label/2024/216158s000lbl.pdf.

[brb371182-bib-0041] Huhn, M. , A. Nikolakopoulou , J Schneider‐Thoma,. et al. 2020. “Comparative Efficacy and Tolerability of 32 Oral Antipsychotics for the Acute Treatment of Adults with Multi‐Episode Schizophrenia: A Systematic Review and Network Meta‐Analysis.” Focus: Journal of Life Long Learning in Psychiatry 18, no. 4: 443. 10.1176/APPI.FOCUS.18306. 33343258 PMC7725155

[brb371182-bib-0042] Kadakia, A. , M. Catillon , Q. Fan , et al. 2022. “The Economic Burden of Schizophrenia in the United States.” The Journal of clinical psychiatry 83, no. 6: 22m14458. 10.4088/JCP.22m14458. 36244006

[brb371182-bib-0043] “KarXT | ALZFORUM .” Accessed: Mar. 20, 2025. [Online]. Available: https://www.alzforum.org/therapeutics/karxt.

[brb371182-bib-0044] “KarXT in Psychosis Associated With Alzheimer's Disease—Clinical Trials Registry—ICH GCP .” Accessed: Feb. 22, 2025. [Online]. Available: https://ichgcp.net/clinical‐trials‐registry/NCT06126224?utm_source=chatgpt.com.

[brb371182-bib-0045] “KarXT in Psychosis Associated With Alzheimer's Disease—Clinical Trials Registry—ICH GCP .” Accessed: Feb. 22, 2025. [Online]. Available: https://clinicaltrials.gov/study/NCT05980949#study‐overview.

[brb371182-bib-0046] Kaul, I. , S. Sawchak , C. U. Correll , et al. 2024. “Efcacy and Safety of the Muscarinic Receptor Agonist KarXT (Xanomeline‐Trospium) in Schizophrenia (EMERGENT‐2) in the USA: Results From a Randomised, Double‐Blind, Placebo‐Controlled, Fexible‐Dose Phase 3 Trial.” Lancet 403, no. 10422: 160–170. 10.1016/s0140-6736(23)02190-6. 38104575

[brb371182-bib-0047] Kaul, I. , S. Sawchak , D. P. Walling , et al. 2024. “Efficacy and Safety of Xanomeline‐Trospium Chloride in Schizophrenia: A Randomized Clinical Trial.” JAMA psychiatry 81, no. 8: 749–756. 10.1001/jamapsychiatry.2024.0785. 38691387 PMC11063924

[brb371182-bib-0048] Kingwell, K. 2024. “FDA Approves First Schizophrenia Drug With New Mechanism of Action Since 1950s.” Nat Rev Drug Discovery 23, no. 11: 803. 10.1038/D41573-024-00155-8. 39333712

[brb371182-bib-0049] Kramer . Brannan, S. K. , C. Sauder , and I Kaul,. 2025. “Safety and Efficacy of Karxt in Patients with Schizophrenia in the Randomized, Double‐Blind, Placebo‐Controlled Emergent Trials.” International Journal of Neuropsychopharmacology 28, no. 1: i162. 10.1093/ijnp/pyae059.279.

[brb371182-bib-0050] Leber, A. , R. Ramachandra , F. Ceban , et al. 2024. “Efficacy, Safety, and Tolerability of Xanomeline for Schizophrenia Spectrum Disorders: A Systematic Review.” Expert Opinion on Pharmacotherapy 25, no. 4: 467–476. 38515004 10.1080/14656566.2024.2334424

[brb371182-bib-0051] Livingston, G. , A. Sommerlad , V. Orgeta , et al. 2017. “Dementia Prevention, Intervention, and Care.” Lancet (London, England) 390, no. 10113: 2673–2734. 10.1016/S0140-6736(17)31363-6. 28735855

[brb371182-bib-0052] Matsunaga, S. , T. Kishi , and N. Iwata . 2015. “Memantine Monotherapy for Alzheimer's Disease: A Systematic Review and Meta‐Analysis.” PLoS ONE 10, no. 4: e0123289. 10.1371/journal.pone.0123289. 25860130 PMC4393306

[brb371182-bib-0053] Mirza, N. R. , D. Peters , and R. G. Sparks . 2003. “Xanomeline and the Antipsychotic Potential of Muscarinic Receptor Subtype Selective Agonists.” CNS Drug Reviews 9, no. 2: 159–186. 10.1111/j.1527-3458.2003.tb00247.x. 12847557 PMC6741650

[brb371182-bib-0054] “Much Ado About Something: Why There's Excitement Behind Novel Mechanism of Action in Schizophrenia .” Accessed: Mar. 20, 2025. [Online]. Available: https://www.psychiatrictimes.com/view/much‐ado‐about‐something‐excitement‐behind‐novel‐mechanism‐action‐schizophrenia?utm_source=chatgpt.com.

[brb371182-bib-0055] Murray, P. S. , S. Kumar , M. A. Demichele‐Sweet , and R. A. Sweet . 2014. “Psychosis in Alzheimer's Disease.” Biological psychiatry 75, no. 7: 542–552. 10.1016/j.biopsych.2013.08.020. 24103379 PMC4036443

[brb371182-bib-0056] Nathan, P. J. , S. B. Millais , A. Godwood , et al. 2022. “A Phase 1b/2a Multicenter Study of the Safety and Preliminary Pharmacodynamic Effects of Selective Muscarinic M 1 Receptor Agonist HTL0018318 in Patients With Mild‐to‐Moderate Alzheimer's Disease.” Alzheimer's and Dementia: Translational Research and Clinical Interventions 8, no. 1. 10.1002/trc2.12273. PMC8864442 35229025

[brb371182-bib-0057] “Psychosis: The Other Big Alzheimer's Therapeutic Target—BioSpace .” Accessed: Jan. 31, 2025. [Online]. Available: https://www.biospace.com/alzheimer‐s‐related‐psychosis‐the‐other‐big‐therapeutic‐target.

[brb371182-bib-0058] Rognoni, C. , A. Bertolani , and C. Jommi . 2021. “Second‐Generation Antipsychotic Drugs for Patients With Schizophrenia: Systematic Literature Review and Meta‐Analysis of Metabolic and Cardiovascular Side Effects.” Clinical drug investigation 41, no. 4: 303–319. 10.1007/s40261-021-01000-1. 33686614 PMC8004512

[brb371182-bib-0059] Saddichha, S. , S. Ameen , and S. Akhtar . 2008. “Predictors of Antipsychotic‐Induced Weight Gain in First‐Episode Psychosis: Conclusions From a Randomized, Double‐Blind, Controlled Prospective Study of Olanzapine, Risperidone, and Haloperidol.” Journal of clinical psychopharmacology 28, no. 1: 27–31. 10.1097/jcp.0b013e3181602fe6. 18204337

[brb371182-bib-0060] Saha, K. B. , L. Bo , S. Zhao , J. Xia , S. Sampson , and R. U. Zaman . 2016. “Chlorpromazine Versus Atypical Antipsychotic Drugs for Schizophrenia.” The Cochrane database of systematic reviews 4, no. 4: CD010631. 10.1002/14651858.CD010631.pub2. 27045703 PMC7081571

[brb371182-bib-0061] Schizophrenia . https://www.who.int/news‐room/fact‐sheets/detail/schizophrenia.

[brb371182-bib-0062] Schmidt, T. , R. Widmer , A. Pfeiffer , and H. Kaess . 1994. “Effect of the Quaternary Ammonium Compound Trospium Chloride on 24 Hour Jejunal Motility in Healthy Subjects.” Gut 35, no. 1: 27–33. 10.1136/gut.35.1.27. 8307445 PMC1374627

[brb371182-bib-0063] Serafini, G. , P. Calcagno , D. Lester , P. Girardi , M. Amore , and M Pompili . 2016. “Suicide Risk in Alzheimer's Disease: A Systematic Review.” Current Alzheimer Research 13, no. 10: 1083–1099. 10.2174/1567205013666160720112608. 27449996

[brb371182-bib-0064] Shin, J. H. , M. F. Adrover , J. Wess , and V. A. Alvarez . 2015. “Muscarinic Regulation of Dopamine and Glutamate Transmission in the Nucleus Accumbens.” Proceedings of the National Academy of Sciences 112, no. 26: 8124–8129. 10.1073/pnas.1508846112. PMC4491757 26080439

[brb371182-bib-0065] Sinclair, L. I. , A. Kumar , T. Darreh‐Shori , and S. Love . 2019. “Visual Hallucinations in Alzheimer's disease Do Not Seem to be Associated With Chronic Hypoperfusion of to Visual Processing Areas V2 and V3 But May be Associated With Reduced Cholinergic Input to These Areas.” Alzheimer's Research and Therapy 11: 80. 10.1186/s13195-019-0519-7. PMC6740037 31511061

[brb371182-bib-0066] Singh, A. 2022. “Xanomeline and Trospium: a Potential Fxed Drug Combination (FDC) for Schizophrenia—A Brief Review of Current Data.” Innov Clin Neurosci 19, no. 10–12: 43–47. 36591549 PMC9776782

[brb371182-bib-0067] Smith, E. E. , N. A. Phillips , H. H. Feldman , et al. 2025. “Use of lecanemab and donanemab in the Canadian Healthcare System: Evidence, Challenges, and Areas for Future Research.” The journal of prevention of Alzheimer's disease 12, no. 3: 100068. 10.1016/j.tjpad.2025.100068. PMC12184013 39893139

[brb371182-bib-0068] Staskin, D. , G. Kay , C. Tannenbaum , et al. 2010. “Trospium Chloride Has no Effect on Memory Testing and is Assay Undetectable in the Central Nervous System of Older Patients With Overactive Bladder.” International journal of clinical practice 64, no. 9: 1294–1300. 10.1111/j.1742-1241.2010.02433.x. 20561092

[brb371182-bib-0069] “Study Details | A Study to Assess Safety and Efficacy of KarXT in Adult Patients With Schizophrenia | ClinicalTrials.gov .” Accessed: Feb. 22, 2025. [Online]. Available: https://www.clinicaltrials.gov/study/NCT03697252.

[brb371182-bib-0070] “Therapeutic Areas | BMS Science | HCP Site .” Accessed: Feb. 23, 2025. [Online]. Available: https://www.bmsscience.com/therapeutic‐area/neuroscience/alzheimers‐disease/?mol=karxt&nct=NCT05511363.

[brb371182-bib-0071] Tricco, A. C. , H. M. Ashoor , C. Soobiah , et al. 2018. “Comparative Effectiveness and Safety of Cognitive Enhancers for Treating Alzheimer's Disease: Systematic Review and Network Metaanalysis.” Journal of the American Geriatrics Society 66, no. 1: 170–178. 10.1111/jgs.15069. 29131306

[brb371182-bib-0072] Tsang, S. W. , P. T. Francis , M. M. Esiri , P. T. Wong , C. P. Chen , and M. K. Lai . 2008. “Loss of [3H]4‐DAMP Binding to Muscarinic Receptors in the Orbitofrontal Cortex of Alzheimer's disease Patients With Psychosis.” Psychopharmacology 198, no. 2: 251–259. 10.1007/s00213-008-1124-9. 18373228

[brb371182-bib-0073] “UW Clinical Trials Institute | New schizophrenia drug could treat Alzheimer's disease .” Accessed: Feb. 17, 2025. [Online]. Available: https://uwclinicaltrials.org/2024/11/21/new‐schizophrenia‐drug‐could‐treat‐alzheimers‐disease/.

[brb371182-bib-0074] van der Westhuizen, E. T. , K. H. C. Choy , C. Valant , et al. 2021. “Fine Tuning Muscarinic Acetylcholine Receptor Signaling Through Allostery and Bias.” Frontiers in Pharmacology 11: 606656. 10.3389/fphar.2020.606656. 33584282 PMC7878563

[brb371182-bib-0075] Vasiliu, O. , B. Budeanu , and M. Ștefan Cătănescu . 2024. “The New Horizon of Antipsychotics Beyond the Classic Dopaminergic Hypothesis—The Case of the Xanomeline–Trospium Combination: A Systematic Review.” Pharmaceuticals 17, no. 5: 610. 10.3390/PH17050610/S1. 38794180 PMC11124398

[brb371182-bib-0076] Vuocolo, S. , W. P. Horan , A. Claxton , et al. 2025. “Efficacy of Karxt on Negative Symptoms in Acute Schizophrenia: an Analysis of Pooled Data from 3 Trials.” International Journal of Neuropsychopharmacology 28, no. 1: i358–i359. 10.1093/IJNP/PYAE059.636.

[brb371182-bib-0077] Weiden, P. J. , A. Breier , S. Kavanagh , A. C. Miller , S. K. Brannan , and S. M. Paul . 2022. “Antipsychotic Efcacy of KarXT (Xanomeline‐Trospium): Post Hoc Analysis of Positive and Negative Syndrome Scale Categorical Response Rates, Time Course of Response, and Symptom Domains of Response in a Phase 2 Study.” Journal of Clinical Psychiatry 83, no. 3. 10.4088/JCP.21m14316. 35552528

[brb371182-bib-0078] Wong, W. 2020. “Economic Burden of Alzheimer Disease and Managed Care Considerations.” The American journal of managed care 26, no. 8: S177–S183. 10.37765/ajmc.2020.88482. 32840331

[brb371182-bib-0079] Yiannopoulou, K. G. , and S. G. Papageorgiou . 2020. “Current and Future Treatments in Alzheimer Disease: An Update.” Journal of central nervous system disease 12: 1179573520907397. 10.1177/1179573520907397. 32165850 PMC7050025

[brb371182-bib-0080] Yohn, S. E. , P. J. Weiden , C. C. Felder , and S. M. Stahl . 2022. “Muscarinic Acetylcholine Receptors for Psychotic Disorders: Bench‐Side to Clinic.” Trends in Pharmacological Sciences 43, no. 12: 1098–1112. 10.1016/j.tips.2022.09.006. 36273943

[brb371182-bib-0081] Zhang, W. , M. Yamada , J. Gomeza , A. S. Basile , and J. Wess . 2002. “Multiple Muscarinic Acetylcholine Receptor Subtypes Modulate Striatal Dopamine Release, as Studied With M1‐M5 Muscarinic Receptor Knock‐Out Mice.” The Journal of Neuroscience : The official journal of the Society for Neuroscience 22, no. 15: 6347–6352. 10.1523/JNEUROSCI.22-15-06347.2002. 12151512 PMC6758135

